# Necrostatin-1 Attenuates Cisplatin-Induced Nephrotoxicity Through Suppression of Apoptosis and Oxidative Stress and Retains Klotho Expression

**DOI:** 10.3389/fphar.2018.00384

**Published:** 2018-04-19

**Authors:** Yichun Ning, Yiqin Shi, Jing Chen, Nana Song, Jieru Cai, Yi Fang, Xiaofang Yu, Jun Ji, Xiaoqiang Ding

**Affiliations:** ^1^Department of Nephrology, Zhongshan Hospital, Fudan University, Shanghai, China; ^2^Shanghai Medical Center for Kidney, Shanghai, China; ^3^Shanghai Key Laboratory of Kidney and Blood Purification, Shanghai, China; ^4^Shanghai Institute of Kidney and Dialysis, Shanghai, China

**Keywords:** cisplatin, Necrostatin-1, necroptosis, apoptosis, inflammation, oxidative stress, Klotho

## Abstract

**Aim:** Cisplatin is an effective chemotherapeutic drug, but the application in clinical is greatly limited by its nephrotoxicity. Necrostatin-1 (Nec-1), an inhibitor of RIP1 kinase, has been reported to inhibit RIP-mediated necroptosis. The aim of this study is to detect the protective effects of Nec-1 on the nephrotoxicity of cisplatin and to investigate its renoprotection mechanism.

**Methods:** 8-week-old male C57BL/6 mice were randomly assigned into four groups: Control, Nec-1, Cisplatin, and Cisplatin+Nec-1. Mice were treated with cisplatin with or without Nec-1 pre-treatment. Renal function, histological changes, necroptosis, and apoptotic markers were investigated. NFκB pathway related proteins, proinflammatory cytokines, oxidative stress markers, renal Klotho, and autophagy-related proteins levels were also examined.

**Results:** Renal function and histological data displayed that the treatment with Nec-1 significantly attenuates cisplatin-induced renal damage. The expression of RIPK1/RIPK3/MLKL were significantly enhanced in cisplatin group as compared to the control group (*p* < 0.05) and was significantly reduced by pre-treatment of Nec-1 (*p* < 0.05). The level of stress and apoptosis-related protein, including p-JNK, p-c-Jun, p-p38, Bax/Bcl-2 ratio, and caspase-3 showed the similar trend. Pre-treatment with Nec-1 inhibit NFκB signaling, reduced proinflammatory cytokines and oxidative stress, up-regulated renal Klotho, and autophagy-related proteins levels.

**Conclusion:** Our results suggest that Nec-1 could be a potential therapeutic drug against the cisplatin-induced nephrotoxicity through its anti-necroptosis, anti-apoptotic, anti-inflammatory anti-oxidant and retain Klotho expression and activate autophagy effects in the kidney.

## Introduction

Cisplatin is quite an effective chemotherapeutic agent and widely used in the treatment of many types of solid organ malignancies. However, the induction of acute kidney injury (AKI) is one of its main adverse effects (Deng et al., 2017). Approximately 25% to 30% of patients experience renal dysfunction after a single dose of cisplatin, which becomes a major limitation to optimal dosing regimens (Lebwohl and Canetta, 1998). Cisplatin acts mainly in proximal tubule epithelial cells of the nephron and induces cell death and inflammation (Faubel et al., 2004; Xu et al., 2015). In recent years, necroptosis, a type of programmed necrosis, plays an important role in cisplatin-induced nephrotoxic AKI (Xu et al., 2015). In addition to its direct tubular toxicity, multiple cell death pathways and a role of inflammation in the pathophysiologic mechanism appear to be involved in cisplatin nephrotoxicity, but the underlying mechanisms are still largely unknown.

Necroptosis, which happens as a result of death receptor signaling upon assembly of the RIP1/RIP3/MLKL necrosome, is involved in various pathologic conditions, including stroke (You et al., 2008), myocardial infarction (Luedde et al., 2014), and the process of ischemia–reperfusion (I/R) injury, such as organ graft I/R injury (Lau et al., 2013; Linkermann and Green, 2014; Pavlosky et al., 2014). The kinase activity of RIP1 was eventually found to be critical for mediating programmed necrosis, the caspase-independent necrotic cell death pathway. In 2005, Degterev and colleagues identified that the 5-(1H-indol-3-ylmethyl)-2-thiohydantoin 1, called Necrostatin-1 (Nec-1), could specifically inhibit RIP1 kinase and then inhibit RIP-mediated necroptosis (Degterev et al., 2005, 2008).

Recent study has further involved the correlation of necroptosis in AKI, including renal I/R injury, cisplatin-induced AKI, and contrast-induced AKI (Linkermann et al., 2012, 2013; Xu et al., 2015; Muller et al., 2017). Nec-1 has been used extensively both *in vitro* and *in vivo* to treat necroptosis-associated diseases. Nec-1 has been shown to protect human proximal tubular cells treated with cisplatin (Tristão et al., 2012). Moreover, Nec-1 attenuated the deterioration of renal morphology in a mouse model of cisplatin-induced AKI (Linkermann et al., 2011). Here we studied how Nec-1 acts in the pathologic process of cisplatin-induced AKI. Consistent with previous research, we found that Nec-1 indeed protected mice from cisplatin-induced nephrotoxicity. We also explored the possible mechanisms of the protection effect of Nec-1, such as inflammation, oxidative stress, renal klotho, and autophagy levels.

## Materials and Methods

### Reagents

Two reagents were used in this study: cisplatin [cisplatinum(II)-diamine dichloride; Sigma, St. Louis, MO, United States] and Nec-1 (Sigma, St. Louis, MO, United States).

### Animals

Eight-week-old (20–25 g) Male C57BL/6 mice (Animal Center of Fudan University, Shanghai, China) were housed in a temperature- and humidity-controlled room on a 12-h light/dark cycle, with standard food and water available *ad libitum*. The study protocol was approved by the Institutional Animal Care and Use Committee of Fudan University, and performed according to the NIH Guide for the Care and Use of Laboratory Animals. The 32 mice were divided into four groups, *n* = 8 per group: (1) control group, (2) Nec-1 group, (3) cisplatin group and (4) cisplatin+Nec-1 group. Mice of the cisplatin group were intraperitoneally injected with a single dose of cisplatin at 20 mg/kg. All mice received 250 μl total volume of normal saline with DMSO or 1.65 mg/kg Nec-1 30 min before the injection of cisplatin. All groups with Nec-1 required repeated Nec-1 injections every 24 h, while all comparison groups received repeated injections of 250 μl of saline with DMSO. Under general anesthesia, mice were sacrificed by exsanguination and the kidneys were harvested at 72 h after the cisplatin injection.

### Kidney Function Assessment

Serum creatinine (Scr) concentration was measured using QuantiChrom^TM^ Creatinine Assay Kit (BioAssay Systems, Hayward, CA, United States) according to the manufacturer’s instructions.

### Histological Analysis

Renal tissue specimens were paraffin embedded, sectioned at 4 μm and submitted to Periodic acid-Schiff (PAS) staining. The analysis was performed by two experienced renal pathologists who were blinded to the study protocol under a light microscopy (Leica DM 6000 B; Leica Microsystems, Wetzler, Germany). For semiquantitative analysis of the frequency and severity of the renal injury, 10 high-magnification (×200) fields of the cortex and the outer stripe of the outer medulla were randomly selected. The specimens were scored according to the extent of foamy degeneration and detachment of tubular cells with the following semiquantitative scale (Racusen and Solez, 1986): no injury (0); mild: <25% (1); moderate: <50% (2); severe: <75% (3); and very severe: >75% (4).

### Immunofluorescence (IF) Staining

Kidney tissues were embedded in OCT compound. 4 μm cryostat sections were stained with anti-RIPK1/RIPK3/MLKL antibody (Abcam, Cambridge, United Kingdom) at 1:100. Sections were incubated sequentially with rhodamine-conjugated AffiniPure goat anti-rabbit immunoglobulin G (IgG) followed by fluorescein-conjugated AffiniPure goat anti-mouse IgG (Jackson ImmunoResearch Laboratories, West Grove, PA, United States). The results were analyzed under a fluorescence microscopy (Olympus BX51, Japan).

### Immunohistochemistry (IHC) Staining

Five-micrometer tissue sections were deparaffinized and rehydrated in graded alcohol and analyzed by the streptavidin-immunoperoxidase technique. Antigen retrieval was carried out with a microwave for 10 min in 0.01 mol/L citrate buffer (pH 6.0). After blocking in 10% normal serum for 30 min, the samples were incubated at 4°C with primary antibodies targeting cleaved caspase-3(Cell Signaling Technology, Danvers, MA, United States, 1:100) and Myeloperoxidase (MPO; Abcam, Cambridge, United Kingdom, 1:100). The specimens were subsequently treated with biotinylated anti-rabbit secondary antibodies for 15 min at 37°C.

### Western Blot

Kidney tissues were homogenized in ice-cold lysis buffer containing protease inhibitor cocktail (Beyotime Biotechnology, China). The supernatant containing the total protein extract was obtained after centrifuging at 12,000 *g* for 30 min at 4°C. The protein concentrations were determined using a BCA Protein Assay kit (KeyGEN Biotech, Nanjing, China). Renal tissue extracts (40 μg/lane) were loaded, separated by SDS-PAGE and transferred onto PVDF membranes (Millipore, Billerica, MA, United States). The membranes were incubated overnight at 4°C with anti-RIPK1/RIPK3/MLKL (Abcam, Cambridge, United Kingdom, 1:1000), p-JNK, p-c-Jun, p-p38, Bax, Caspase-3, p-IKKα/β, IKKα, IKKβ, p-IκBα, IκBα, p-NFκB p65, NFκB p65 (Cell Signaling Technology, Danvers, MA, United States, 1:1000), klotho, Beclin 1, and LC3 II (Abcam, Cambridge, United Kingdom, 1:1000), GAPDH (Abcam, Cambridge, United Kingdom, 1:2000). Next, the membranes were incubated with goat, anti-rabbit, or mouse IgG horse-radish peroxidase conjugate (Jackson ImmunoResearch Inc, United States, 1:5000) and then scanned using LAS-3000 detection system. The band intensities were analyzed using ImageJ software and the densitometric intensity corresponding to each band was normalized against GAPDH expression.

### RNA Isolation and Quantitative Real-Time RT-PCR

The total RNA and cDNA were prepared from whole kidney samples using TRIzol reagent (Sigma Aldrich, St. Louis, MO, United States) and RT-PCR kits (TaKaRa, Japan). Real-time RT-PCR was carried out with SYBR Premix ExTaqTM from TaKaRa. The following primers (Sangon, China) were used: IL-1b forward 5′-ACTACAGGCTCCGAGATGAA-3′ and reverse 5′-TGGGTCCGACAGCACGAGGC-3′; IL-6 forward 5′- GAGGATACCACTCCCAACAGACC-3′ and reverse 5′- AAGTGCATCATCGTTGTTCATACA-3′; TNF-a forward 5′-CCTGGCCAACGGCATGGATC-3′ and reverse 5′-CGGCTGGCACCACTCGTTGG-3′; CCL5 forward 5′-CCCTCACCATCATCCTCACT-3′ and reverse 5′-CCACTTCTTCTCTGGGTTGG-3′; GAPDH forward 5′-TGCACCACCAACTGCTTAG-3′ and reverse 5′-GGATGCAGGGATGATGTTC-3′. Relative mRNA amounts were derived by the 2^(-ΔΔC_T)_^ method.

### Measurement of Malondialdehyde (MDA) and Myeloperoxidase (MPO) Levels

MDA content and MPO activity in kidney homogenate were measured by biochemical assay kits (Nanjing Jiancheng, Nanjing, China) according to the manufacturer’s instructions (Ye et al., 2015).

### Statistical Analysis

Results are presented as mean ± standard deviation (SD). SPSS 19.0 (SPSS Inc., United States) was used for statistical analyses. One-way analysis of variance (ANOVA) and Student’s *t*-test were employed to assess differences among and between groups, respectively. Statistical significance of difference was defined as a *p* < 0.05.

## Results

### Nec-1 Attenuates Cisplatin-Induced Nephrotoxicity

**FIGURE 1:**
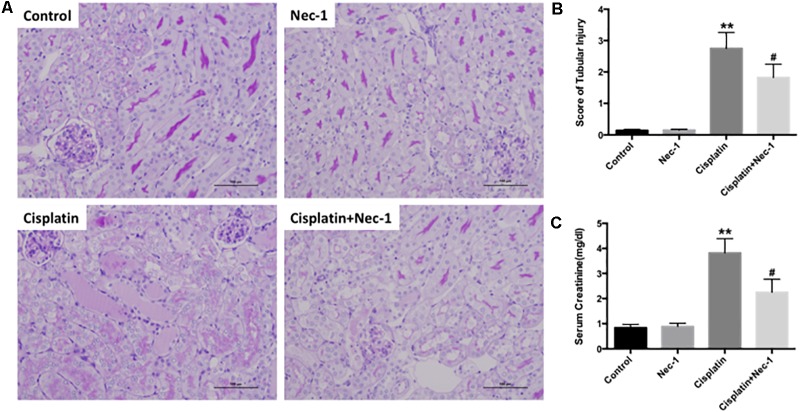
Nec-1 attenuates the cisplatin-induced nephrotoxicity in mice. **(A)** Representative photomicrographs of renal tissues submitted to PAS staining (×200). **(B)**Tubular injury scores provided semi-quantitative data of the morphological changes by histological grading system. **(C)** Changes of serum creatinine among the four groups. The data are presented as means ±*SD* (*n* = 8). ^∗∗^*p* < 0.01 versus control group; #*p* < 0.05 versus cisplatin group.

To confirm the protective effect of Nec-1 on renal damage after cisplatin injection, we determined the histopathologic evaluation. Compared to the control or Nec-1 alone group, at 72 h after cisplatin treatment, PAS-stained kidney tissues exhibited extensive tubular damage, including tubular cell necrosis, the PAS-positive material accumulation in the tubular lumen, brush-border membrane loss, tubular dilatation, and inflammatory cell infiltration (**Figures 1A,B**; ^∗∗^*p* < 0.01). However, the tubular injury was reduced by Nec-1 treatment, which was presented as lower tubular injury score (**Figure 1B**; #*p* < 0.05). Elevation of Scr concentration was significantly reduced in Nec-1 treated mice at 72 h after cisplatin injection (**Figure 1C**; #*p* < 0.05), which was consistent with the histopathologic evaluation. Treatment with Nec-1 alone caused no significant morphologic alterations and renal function.

### Both Necroptosis and Apoptosis Pathway Involved in the Renoprotection of Nec-1

**FIGURE 2:**
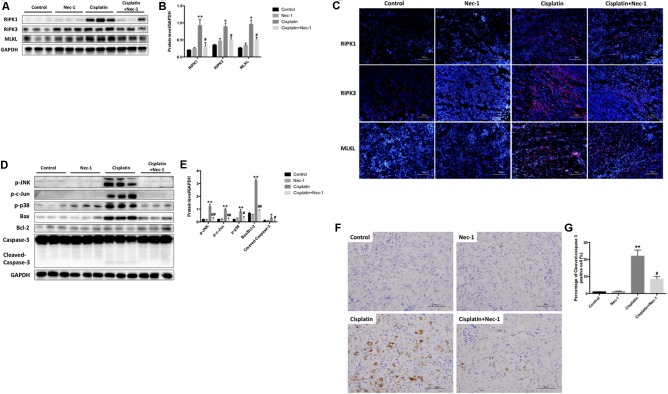
Both necroptosis and apoptosis pathway involved in the renoprotection of Nec-1. **(A,B)** The protein levels of RIP1/RIP3/MLKL were all significantly increased in the kidneys of the cisplatin group compared with the control (^∗∗^*p* < 0.01, ^∗^*p* < 0.05 versus control group) and Nec-1 groups and was significantly reduced by plus Nec-1 treatment (#*p* < 0.05 versus cisplatin group). **(C)** IF staining indicated that the positive areas of RIP1/RIP3/MLKL were mainly located in tubular epithelial cells from kidneys after cisplatin administration. **(D,E)** The levels of stress and apoptosis-related protein, including p-JNK, p-c-Jun, p-p38, Bax/Bcl-2 ratio, and caspase-3, were significant in the kidneys at 72 h after cisplatin treatment (^∗∗^*p* < 0.01, ^∗^*p* < 0.05 versus control group). However, these effects were eliminated in different level after Nec-1 administration (##*p* < 0.01, #*p* < 0.05 versus cisplatin group). **(F,G)** IHC staining of cleaved caspase-3 showed the same tendency among the four groups (^∗∗^*p* < 0.01 versus control group, #*p* < 0.05 versus cisplatin group).

To explore induction of necroptosis in the cisplatin-induced AKI, we detected the necroptosis-related protein (RIPK1, RIPK3, and MLKL) levels in kidneys at 72 h after cisplatin treatment using WB analysis. As shown in **Figures 2A,B**, the level of RIPK1 was significantly enhanced in the cisplatin group as compared to the control group (^∗∗^*p* < 0.01) and was significantly reduced with Nec-1 treatment (#*p* < 0.05). The expressions of RIPK3 and MLKL increased simultaneously with RIPK1 in the cisplatin group and the similar trend was also observed in the Nec-1 treatment group. Consistent with the WB results, IF analysis further corroborated the increase of necroptosis-related protein levels after cisplatin treatment at 72 h. IF analysis showed that RIPK1/RIPK3/MLKL staining positive areas were mainly located in tubular epithelial cells from the cisplatin-induced kidneys. By plus Nec-1 treatment the positive area of RIPK1/RIPK3/MLKL was reduced (**Figure 2C**).

Numerous research indicate that tubular cell apoptosis and MAPK signaling exacerbate the pathogenesis of cisplatin-induced AKI. Thus, we determined the role of Nec-1 in attenuating the extent of cisplatin-induced tubular epithelial cell stress and apoptosis in mice. Using WB analysis of kidney extracts, we detected the levels of stress and apoptosis-related protein, including p-JNK, p-c-Jun, p-p38, Bax/Bcl-2 ratio, and caspase-3. Consistent with other studies, we found that these apoptosis pathway proteins were significantly activated in kidneys at 72 h after cisplatin treatment (**Figures 2D,E**; ^∗^*p* < 0.05, ^∗∗^*p* < 0.01). However, these effects were eliminated in different level after Nec-1 administration (#*p* < 0.05, ##*p* < 0.01). Meanwhile, IHC staining of cleaved caspase-3 showed the same tendency among the four groups (**Figures 2F,G**).

### Nec-1 Reduces Inflammation in Cisplatin-Induced Nephrotoxicity

**FIGURE 3:**
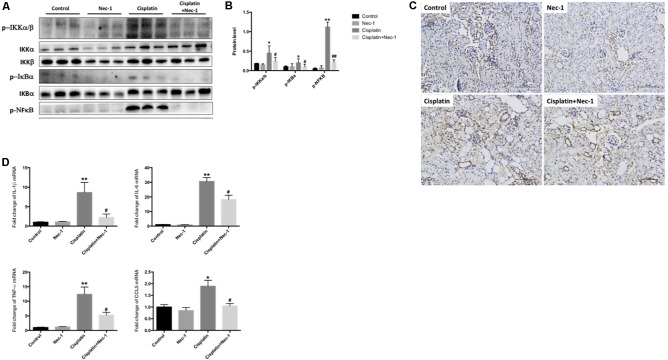
Nec-1 reduces inflammation in cisplatin-induced nephrotoxicity. **(A,B)** The NFκB signaling related proteins of p-IKKα/β, p-IκB, and p-NFκB p65 tested by western blotting were activated in the cisplatin-induced group (^∗^*p* < 0.05, ^∗∗^*p* < 0.01 versus control group). The administration of Nec-1 significantly ameliorated these effects following cisplatin insult (#*p* < 0.05, ##*p* < 0.01 versus cisplatin group). **(C)** IHC staining showed that the positive areas for NFκB were mainly located in tubular epithelial cells from the cisplatin-induced kidneys. **(D)** The proinflammatory cytokines mRNA levels of IL-1β, IL-6, TNF-α, and CCL5 tested by real-time RT-PCR were much greater in the cisplatin group compared with the control group (^∗^*p* < 0.05, ^∗∗^*p* < 0.01). The administration of Nec-1 strongly ameliorated inflammatory factors (#*p* < 0.05 versus cisplatin group).

NFκB transcription factor has been regarded the central mediator of the inflammatory process. To determine whether Nec-1 could attenuate the inflammation induced by cisplatin and to explore the molecular mechanisms underlying the anti-inflammatory role of Nec-1, we next investigated NFκB signaling related proteins of p-IKKα/β, p-IκB, and p-NFκB p65 by western blotting and the proinflammatory cytokines mRNA levels of IL-1β, IL-6, TNF-α, and CCL5 by real-time RT-PCR. In **Figures 3A,B**, the results of WB showed that NFκB signaling were activated in the cisplatin group (^∗^*p* < 0.05, ^∗∗^*p* < 0.01). The administration of Nec-1 significantly ameliorated these effects following cisplatin insult (#*p* < 0.05, ##*p* < 0.01). IHC staining indicated that the positive areas for NFκB were mainly located in tubular epithelial cells from the cisplatin-induced kidneys (**Figure 3C**). The mRNA levels of proinflammatory mediators were much greater in the cisplatin group than in the control and Nec-1 groups (**Figure 3D**; ^∗^*p* < 0.05, ^∗∗^*p* < 0.01). Correspondingly, the treatment of Nec-1 strongly alleviated inflammatory factors (#*p* < 0.05).

### Nec-1 Attenuates Oxidative Stress in Cisplatin-Induced Nephrotoxicity

**FIGURE 4:**
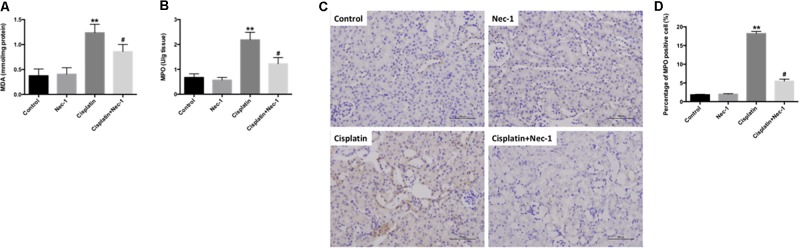
Nec-1 attenuates oxidative stress in cisplatin-induced nephrotoxicity. **(A,B)** The oxidative stress parameters MDA content and MPO activity at 72 h after cisplatin administration in the kidneys were significantly increased (^∗∗^*p* < 0.01 versus control group). Nec-1 reduced the upregulation of MDA and MPO levels (#*p* < 0.05 versus cisplatin group). **(C,D)** IHC staining of MPO in kidneys showed the similar trend among the four groups (^∗∗^*p* < 0.01 versus control group, #*p* < 0.05 versus cisplatin group).

ROS and oxidative damage are very important factors in cisplatin-induced AKI. Several researchers also have shown that ROS are closely related to nephrotoxicity induced by cisplatin. Therefore, we measured principal oxidative stress parameters including MDA content and MPO activity at 72 h after cisplatin administration in the kidneys. We found that the levels of MDA and MPO were significantly increased in kidneys of mice following cisplatin injection (**Figures 4A,B**; ^∗∗^*p* < 0.01). However, Nec-1 can reduce the upregulation of MDA and MPO levels (#*p* < 0.05). Furthermore, the IHC staining of MPO in kidneys also confirmed this result (**Figures 4C,D**; ^∗∗^*p* < 0.01, #*p* < 0.05).

### Nec-1 Retains Renal Klotho and Autophagy-Related Protein Levels

**FIGURE 5:**
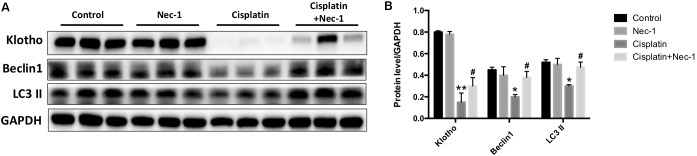
Nec-1 retains renal Klotho and autophagy-related proteins levels. **(A,B)** The renal Klotho, Beclin-1, and LC3-II protein levels were significantly decreased in the kidneys of mice after cisplatin administration (^∗∗^*p* < 0.01, ^∗^*p* < 0.05 versus control group). Nec-1 treatment effectively retained renal Klotho, Beclin-1, and LC3-II levels following cisplatin insult (#*p* < 0.05 versus cisplatin group).

Klotho, a single-pass transmembrane protein, is highly expressed in the kidney. Emerging evidence has revealed that Klotho cannot be only as an early biomarker but also a therapeutic agent for AKI (Hu and Moe, 2012). Recent study has also shown that Klotho has protective effects on cisplatin-induced AKI (Panesso et al., 2014). Thus, we examined whether Nec-1 retains renal Klotho in the mice after cisplatin injection. We found that the Klotho protein level was significantly decreased in the kidneys of cisplatin-treated mice (**Figures 5A,B**; ^∗∗^*p* < 0.01). Nec-1 treatment could effectively retain renal Klotho following cisplatin insult (#*p* < 0.05). In addition, accumulated evidences demonstrated that autophagy may protect against renal tubular cell death induced by cisplatin (Periyasamy-Thandavan et al., 2008; Takahashi et al., 2012; Li et al., 2014). Thus, we assessed the autophagy related proteins such as Beclin-1 and LC3-II using WB analysis. As shown in **Figures 5A,B**, Beclin-1 and LC3-II in the cisplatin group were concurrently decreased compared to the control group (^∗^*p* < 0.05). However, Nec-1 treatment significantly elevated Beclin-1 and LC3-II expression levels compared with the cisplatin group (#*p* < 0.05). These findings suggested that autophagy may play an important role in the therapeutic effect of Nec-1 in cisplatin-induced AKI.

## Discussion

Cisplatin is one of the most important drugs in the chemotherapy of solid tumors. However, the clinical application of cisplatin is limited mostly by its nephrotoxicity. Numerous previous studies reveal that apoptosis and necrosis are major mechanism underlying cisplatin-induced renal cell injury. Apoptosis and necrosis are two important types of pathologies associated with cell death. Apoptosis is considered to be a tightly controlled and regulated cell death during development and in the process of physiological cellular turnover, while necrosis was thought to happen mainly in an uncontrolled manner (Vandenabeele et al., 2010). Necroptosis is currently considered to be another form of procedural death. Necroptosis, which involves the loss of membrane integrity and release of damage-associated molecular pattern molecules (DAMPs), is closely related to inflammatory response (Zhao et al., 2015). Both apoptosis and necroptosis were convincingly demonstrated to contribute to the pathophysiology. Although a recent study reported the protective effects of Nec-1 on cisplatin-induced nephrotoxicity in mice (Xu et al., 2015; Tristão et al., 2016), the relative mechanism and comprehensive explanation of Nec-1 itself on prevention and treatment in mice with cisplatin-induced nephrotoxicity are not yet clear. This study demonstrated that Nec-1 is able to elevate renal function recovery after cisplatin-induced AKI in mice through both necroptosis and apoptosis pathway. We also explored the possible mechanisms of the protection effect of Nec-1, such as inflammation, oxidative stress, renal Klotho, and autophagy levels. These results implied that uninterrupted Nec-1 administration was a guaranteed therapeutic strategy for cisplatin-induced nephrotoxicity.

Necroptosis, a form of programmed necrosis, is executed by RIPK1 and/or RIPK3 when caspases are inhibited (Linkermann and Green, 2014; Galluzzi et al., 2017). More and more evidence has indicated that necroptosis plays an important role in the pathogenesis of different kinds of kidney diseases, including renal I/R injury (Linkermann et al., 2012), cisplatin-induced AKI (Xu et al., 2015), and contrast-induced AKI (Linkermann et al., 2013). Consistent with previous research, we found that Nec-1 indeed protected mice from cisplatin-induced nephrotoxicity. Meanwhile, we tested tubular cell apoptosis and stress signaling in the pathogenesis of cisplatin-induced AKI. MAPK (p38 mitogen-activated protein kinase) and JNK (c-Jun amino terminal kinase) are two major stress-activated protein kinases. Both p38 and JNK pathways highly activation occurs in human renal diseases, such as glomerulonephritis and AKI (Ma et al., 2009). Application of small molecule inhibitors of p38 and JNK has been shown to inhibit renal inflammation, fibrosis, and apoptosis and then protect from renal injury in various kinds of experimental kidney diseases (Ma et al., 2009; Kim et al., 2014). Our study showed that Nec-1 alleviated the degree of tubular epithelial cell stress and apoptosis induced by cisplatin in mice. However, previous studies done by Tristão and colleagues showed that Nec-1’s renoprotection was non-apoptosis related (Tristão et al., 2012, 2016). The doses and injection times of cisplatin and nec-1 used in our study were different with Tristão’s study, and the time of kidney collection was also different. Therefore, the findings of two *in vivo* experiments could be seemingly opposite. Another study in a murine *in vivo* model of myocardial I/R injury found that lower concentrations (30 μM) of Nec-1 protected against myocardial infarction, but raising its concentration to 100 μM enhanced infarct size. It was concluded that at higher concentrations Nec-1 may produce non-specific or toxic actions that potentiate apoptotic and necrotic mechanisms, culminating in enhanced myocardial infarction (Smith et al., 2007). The protective effect of Nec-1 in different disease models can be attributed to inhibition of RIPK1, indoleamine 2,3-dioxygenase, or both. Therefore, in pathological conditions, it cannot a priori be said whether Nec-1 mediated inhibition targets, RIPK1-mediated apoptosis, or RIPK1-mediated necroptosis (Vandenabeele et al., 2013). Nevertheless, we do not know the more in-depth molecular mechanisms. This is also the flaw of this study.

RIPK1 and RIPK3, two key necroptotic effectors, directly initiate or regulate inflammatory signaling pathways. RIPK3 ablation in mice prevented epithelial cell death and inflammation in both the colon and the small intestine. This provides *in vivo* experimental evidence that necroptosis of epithelial cells mediated by RIPK3 induces intestinal inflammation (Welz et al., 2011). Moreover, RIPK3 deficiency relieved skin inflammation (Ma et al., 2009). As expected, the Nec-1 treatment alleviated the inflammation of cisplatin-induced AKI. We found that the central inflammatory process NFκB pathway and proinflammatory mediators such as IL-1β, IL-6, TNF-α, and CCL5 in the pathological process of cisplatin-induced renal injury were ameliorated by Nec-1.

Previous research has already reported that nephrotoxicity induced by cisplatin is closely associated with ROS which leads to oxidative stress (Kim et al., 2010). Oxidative stress generated by cisplatin was considered to be one of the main causes for AKI happening. Oxidative stress leads to lipid peroxidation and the activity of antioxidant defense enzymes decreasing, which leads to cell damage (Antunes et al., 2000; Karadeniz et al., 2011; Deng et al., 2017). Then the damage causes the formation of the harmful product MDA and MPO, which levels increasing is considered to be oxidative stress markers. In the current study, it was observed that MDA and MPO activities were significantly increased in kidneys of mice treated by cisplatin, while Nec-1 can partially reduce the upregulation of MDA and MPO levels. Cisplatin-induced oxidative stress can activate p38, which can increase caspase 3 expression, thereby resulting in cells apoptosis (Miller et al., 2010; Ravindran et al., 2011; Khan et al., 2012). In this study, we found that the levels of p-p38 and cleaved caspase 3 in cisplatin-treated group were higher than the control group, while the above effect was significantly reduced after Nec-1 application. These results further support oxidative stress involved in cisplatin-induced acute renal injury. It is one of the foundations for clinical treatment of cisplatin-induced nephrotoxicity.

Klotho, a single-pass transmembrane protein, was originally identified as an important contributor to lifespan in 1997 (Kuro-o et al., 1997). In the mammalian kidney, Klotho is highly expressed in distal convoluted tubules and is also found in the proximal convoluted tubule, although at lower levels (Hu et al., 2010). Renal Klotho deficiency is found in many types of AKI such as that induced by ureteral obstruction (Doi et al., 2011), lipopolysaccharide (model of sepsis) (Ohyama et al., 1998), nephrotoxins including cisplatin (Panesso et al., 2014), and folic acid (Moreno et al., 2011). Ample data demonstrate that Klotho can be as a biomarker and therapy agent for AKI. In this study, we found that Nec-1 retains renal Klotho in the mice following cisplatin insult. Autophagy appears to be a downstream event following Klotho activation (Shu et al., 2013). In addition, accumulated evidence demonstrated that autophagy may protect against tubular cell death induced by cisplatin (Periyasamy-Thandavan et al., 2008; Takahashi et al., 2012; Li et al., 2014). It is observed that autophagy related proteins such as Beclin-1 and LC3-II decreased in cisplatin group and Nec-1 treatment significantly elevated them. However, previous studies found that Nec-1 could suppress Beclin-1 and LC3-II activation, reduce autophagosome formation, and promote autophagosome elimination (Wang et al., 2012; Zhou et al., 2013; Dong et al., 2018). In another study, Nec-1 could not inhibit LC3-II generation induced with obatoclax and dexamethasone treatment in Jurkat cells (Bonapace et al., 2010). These findings indicated that Nec-1 can play different roles in different pathological conditions, and cause different effects and results. The crosstalk between necroptosis and other cell death pathways, such as apoptosis and autophagy, is very complicated. Therefore, it seems reasonable with the result of Nec-1 activating autophagy in cisplatin-induced AKI, although the in-depth mechanism of such result is not yet clear and still needs further investigation.

## Conclusion

Our study demonstrated that Nec-1 had protective effect against cisplatin-induced nephrotoxicity in mice. The renoprotective effect of Nec-1 could be due to the suppression of necroptosis and apoptosis pathways, reduction of inflammation and oxidative stress, restoration of renal Klotho, and autophagy levels. Furthermore, the renoprotective mechanism may also involve attenuation of pro-inflammatory mediators and inhibition of NFκB activation. However, further studies are needed to assess the potential use of Nec-1 as an adjuvant therapy to reduce cisplatin-induced nephrotoxicity in clinical and provide better knowledge of physiological mechanisms for Nec-1 renal protection.

## Author Contributions

YN conceived and designed the experiments. YN and YS performed the experiments. YN and JinC analyzed the data. NS, JieC, XY, YF, and JJ contributed reagents/materials/analysis tools. YN and XD wrote the manuscript.

## Conflict of Interest Statement

The authors declare that the research was conducted in the absence of any commercial or financial relationships that could be construed as a potential conflict of interest.
